# Utilizing Genomics to Characterize the Common Oat Gene Pool—The Story of More Than a Century of Polish Breeding

**DOI:** 10.3390/ijms24076547

**Published:** 2023-03-31

**Authors:** Aneta Koroluk, Sylwia Sowa, Maja Boczkowska, Edyta Paczos-Grzęda

**Affiliations:** 1 Institute of Plant Genetics, Breeding and Biotechnology, University of Life Sciences in Lublin, 20-950 Lublin, Poland; aneta.koroluk@up.lublin.pl (A.K.); sylwia.sowa@up.lublin.pl (S.S.); 2 Plant Breeding and Acclimatization Institute, National Research Institute, 05-870 Radzików, Poland; m.boczkowska@ihar.edu.pl

**Keywords:** *Avena sativa*, oat, genetic diversity, DArTseq, SNP, cultivars, breeding

## Abstract

This study was undertaken to investigate the diversity and population structure of 487 oat accessions, including breeding lines from the ongoing programs of the three largest Polish breeding companies, along with modern and historical Polish and foreign cultivars. The analysis was based on 7411 DArTseq-derived SNPs distributed among three sub-genomes (A, C, and D). The heterogeneity of the studied material was very low, as only cultivars and advanced breeding lines were examined. Principal component analysis (PCA), principal coordinate analysis (PCoA), and cluster and STRUCTURE analyses found congruent results, which show that most of the examined cultivars and materials from Polish breeding programs formed major gene pools, that only some accessions derived from Strzelce Plant Breeding, and that foreign cultivars were outside of the main group. During the 120 year oat breeding process, only 67 alleles from the old gene pool were lost and replaced by 67 new alleles. The obtained results indicate that no erosion of genetic diversity was observed within the Polish native oat gene pool. Moreover, current oat breeding programs have introduced 673 new alleles into the gene pool relative to historical cultivars. The analysis also showed that most of the changes in relation to historical cultivars occurred within the A sub-genome with emphasis on chromosome 6A. The targeted changes were the rarest in the C sub-genome. This study showed that Polish oat breeding based mainly on traditional breeding methods—although focused on improving traits typical to this crop, i.e., enhancing the grain yield and quality and improving adaptability—did not significantly narrow the oat gene pool and in fact produced cultivars that are not only competitive in the European market but are also reservoirs of new alleles that were not found in the analyzed foreign materials.

## 1. Introduction

One of the greatest modern challenges is the rapid growth of the human population, estimated to reach 10 billion over the next 30 years [1]. Hence, there is a need to increase agricultural production to provide global food security. Human nutrition is based on the cereals belonging to the *Poaceae* family, also known as *Gramineae*, of which wheat, corn, and rice provide nearly two-thirds of the global caloric intake [2,3]. Oat (*Avena sativa* L.) significantly stands out from other cereals. Oat grain is rich in vitamins and macro-and microelements and has the lowest polysaccharide content with a predominance of starch. 

Oat contains large amounts of dietary fiber—in particular, its soluble fraction is rich in β-glucans, ingredients that have been one of the greatest discoveries in recent years [4,5]. Of particular interest are oat avenanthramides, reported to possess antioxidant [6] and cholesterol-lowering properties [7]. The extraordinary nutritional value and health-promoting compounds increase the economic importance of oat, extend the requirements for new oat breeding cultivars, and develop new breeding directions.

In 2021, the area in Poland planted with oats occupied 527.405 thousand hectares, placing Poland among the top five oat producers globally, next to Russia, Canada, Australia, and the USA (http://faostat3.fao.org, http://stat.gov.pl/, accessed on 5 January 2023).

Polish oat breeding has a rich history of over 120 years. The beginning of oat breeding on Polish territory dates back to the end of the nineteenth century. The first reports of Polish cultivars appeared as early as 1893 when Antoni Sempołowski, considered the pioneer of Polish breeding, developed the cultivar ‘Sobieszyński’, which was derived from ‘Rychlik Lubelski’, descended from oats grown by peasants in the Lublin region. This confirms that the first Polish oat cultivars were developed even before 1893. Until World War II, around 60 Polish cultivars were bred. During the war, almost all Polish breeding materials were destroyed, and oat breeding had to be thoroughly rebuilt. 

For many years after the war, the Polish oat register was based on the pre-war cultivars from the 1920s. Some of the old varieties, such as Udycz Żółty, due to the high quality comparable to other European oats, were listed in the register up until 1975 [8]. In 1964, due to the lack of new Polish cultivars, the market was mostly dominated by foreign varieties. Since 1977, this monopoly began to be overcome by new, Polish cultivars, of which the Dragon and Markus developed in Danko Plant Breeding Ltd. played a major role [9]. Since then, due to breeding development, many excellent Polish cultivars have emerged, bringing a richness of favorable traits.

In order to achieve new breeding goals, i.e., to increase the yield stability, nutritional value, and resistance to biotic and abiotic factors, a thorough characterization of breeding materials is necessary. Assessing the extent of the genetic diversity among adapted, elite germplasms may be useful for estimating the genetic variability among segregating progeny. In the studies conducted so far, the analyses of the genetic diversity of Polish cultivars gene pool have mainly been based on randomly amplified polymorphic DNA (RAPD), amplified fragment length polymorphism (AFLP), retrotransposon–microsatellite amplified polymorphism (REMAP), and inter simple sequence repeat (ISSR) [10,11,12,13]. 

PCR-based markers have been used to analyze the genetic diversity of 23 primary oat cultivars bred in Poland before 1939 [12], that of 19 oat cultivars registered in Poland in the years 1984–2004 [11], and that of selected modern and old Polish cultivars and landraces [13,14,15]. Even though PCR-based techniques are widely used and affordable, the use of techniques based on large-scale DNA sequencing enables a significant increase in the number of polymorphisms identified in a single experiment. Among the many techniques available, DArT genotyping by sequencing (DArTseq^TM^) is very popular and often used. DArTseq is a highly informative genome marker technology based on next-generation Illumina short-read sequencing [16,17]. 

This technique has enabled the successful genetic diversity evaluation of many species, including *Triticum aestivum* [18], *Allium sativum* [19], *Hordeum vulgare* [20,21], and *Solanum lycopersicum* [22]. In this study, DArTseq-derived SNPs were used to genotype breeding lines from the ongoing programs of the three largest Polish breeding companies, along with modern and historical Polish and foreign cultivars to assess the genetic relatedness and population structure of Polish breeding materials. The material used had never been genotyped by sequencing before, and thus the obtained results may increase breeders’ knowledge of oat germplasm and assist in the selection of materials useful for modern oat-breeding programs. 

## 2. Results

### 2.1. Data Quality

The SNP dataset obtained using the DArTseq^TM^ genome reduction method contained 32,253 markers. The average rate of missing values per SNP marker was 11.36%. To avoid errors in the statistical analysis, loci with more than 5% missing data, low reproducibility (RepAvg ≤ 1), and low minor allele frequency (MAF < 0.01) were removed. Therefore, 24,842 SNP loci were excluded, and 7411 met all quality parameters and were subjected to further analysis.

**Figure 1:**
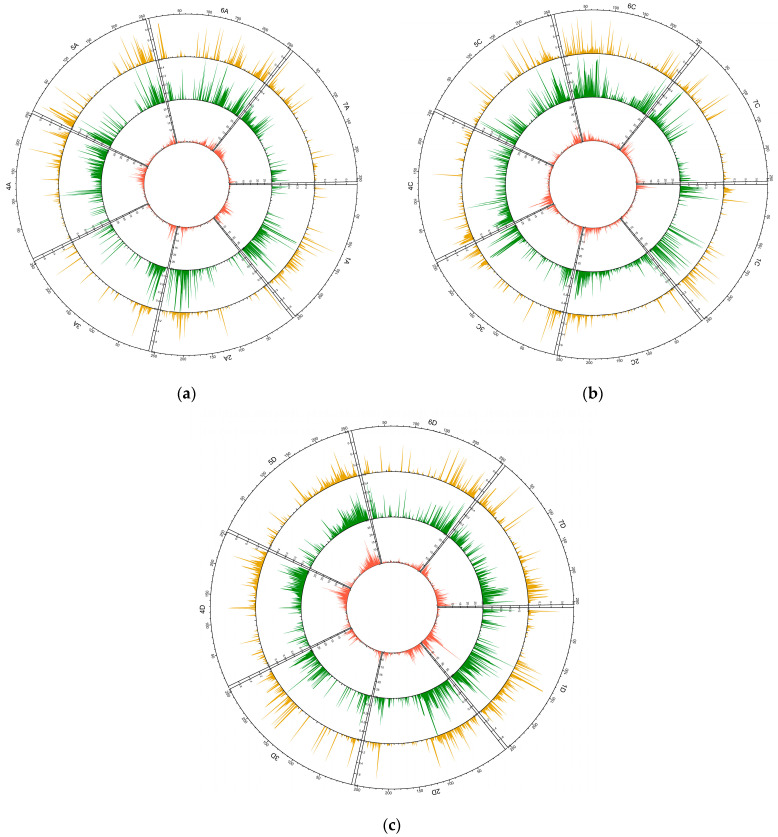
Circular overview of 21 chromosomes of A, C, and D oat reference genome OT3098 [23] for 487 *A. sativa* genotypes based on DArTseq data. (**a**) DArTseq loci distribution; (**b**) average polymorphism information content (PIC) distribution; and (**c**) average observed heterozygosity (Ho) distribution.

Within the remaining SNPs, a total of 6317 had known positions on the chromosomes of A-, C-, and D-reference OT3098 oat genomes v2 [23] (Figure 1). There were 70 SNPs assigned to the unknown chromosome of OT3098 composed of the sequences unassigned to the 21 chromosomes, and 1024 markers not associated with any reference oat chromosome. Most of the loci analyzed were located on chromosome 4D (524), and the least were on 2A (148). In general, the patterns of the studied loci distribution along each chromosome were similar; the number of loci was higher at the ends of chromosomes and decreased towards the centromere.

In the studied oat collection, more transition-type (53.05%) than transversion-type (46.95%) mutations were observed with a transition/transversion (Ts/Tr) SNP rate of 1 > 13 (Table 1). Within the transition, more changes occurred within purines (51.69%) than pyrimidines (48.31%). In the A and C sub-genomes, the most frequent change was A > G, and in the D sub-genome, G > A. For transversions, there were generally more pyrimidine > purine changes (51.01%) than purine > pyrimidine (48.99%), and the most frequently observed mutation was C > G (20.89% of all transversions). In the A and D sub-genomes, the most common mutation type was C > G, and in the C sub-genome, G > C. The least frequent type of change was A > T with only a single mutation of this type observed on chromosome 6D.

**Figure 2:**
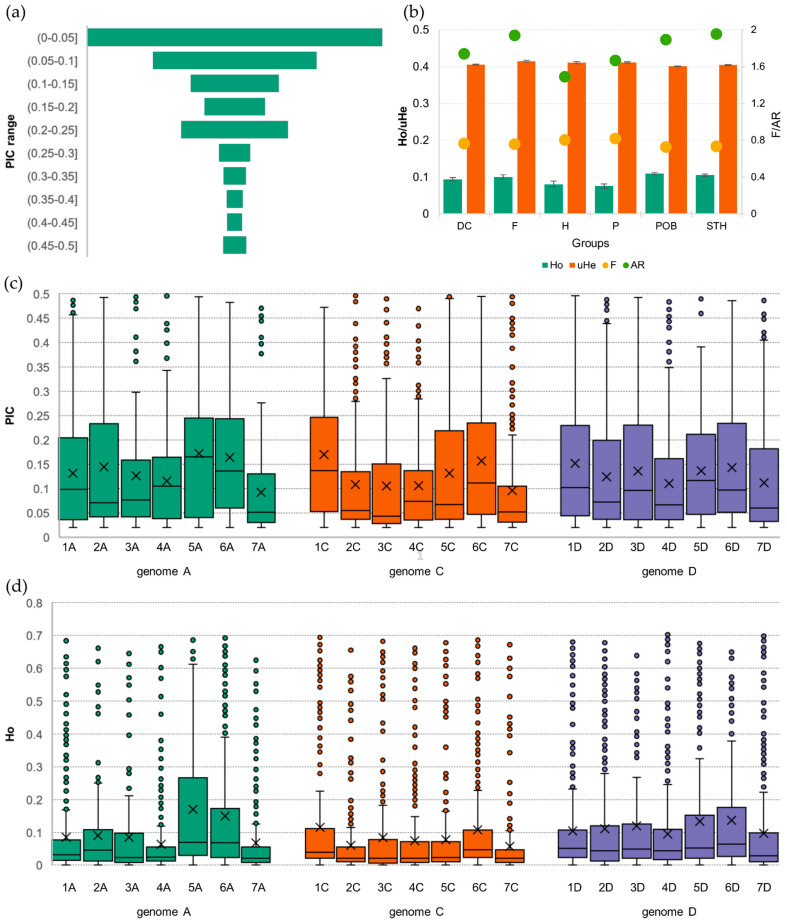
Summary of coefficients for 487 accessions of *A. sativa*; (**a**) polymorphic information content (PIC); (**b**) diversity coefficients: heterogeneity (Ho) and expected heterozygosity (He), inbreeding factor (F) and allelic richness (AR); (**c**) PIC by chromosome; and (**d**) heterogeneity by chromosome.

As a result of the applied raw data filtering parameters, the PIC values ranged from 0.02 to 0.50 with a mean of 0.130 and a median of 0.081 (Figure 1). The mean PIC values in the homoeologous genomes did not differ significantly. In sub-genome A, the highest mean PIC value was observed for chromosome 5A (0.172), and the lowest for 7A (0.092). In the C sub-genome, the highest average PIC was observed for chromosome 1C (0.169), and the lowest for 7C (0.096). In the D sub-genome, the average PIC had a maximum for chromosome 1D (0.152) and a minimum for 4D (0.111). Of the analyzed SNPs, 36% had a PIC not exceeding 0.05, and only about 5% had a PIC above 0.4 (Figure 2).

### 2.2. Genetic Diversity

The average heterozygosity (Ho) in the studied set, which, for the studied material, reflects the heterogeneity of accessions, was very low at 0.10. The proportion of heterozygous loci in each sub-genome showed no significant statistical differences. In sub-genome A, it ranged from 0. 063 (4A) to 0.170 (5A); in sub-genome C, from 0.057 (7C) to 0.115 (1C); and in sub-genome D, from 0.097 (7D) to 0.137 (6D). There were statistically significant differences in the levels of the mean heterogeneity of the groups studied. The maximum Ho value was observed in the POB group (0.109), and the minimum was in the P group (0.075), representing modern Polish cultivars. 

A detailed analysis of the heterogeneity within the groups, i.e., at the chromosome level, showed that the maximum Ho in foreign cultivars (F) and breeding materials from Małopolska Plant Breeding Ltd. (POB), Strzelce Plant Breeding Ltd. (STH), and Danko Plant Breeding Ltd. (DC) occurred on chromosome 5A, and in the modern Polish (P) and historic (H) cultivars, on 6A. In contrast, the most homogeneous loci were found on chromosomes 7C (the DC, F, H, and STH groups) and 4A (the P and POB groups). For nearly every chromosome, there was a group of outlier loci that had a very high level of heterogeneity. 

The mean value of the inbreeding coefficient F in the study set was 0.75. It was the highest in the P group and the lowest in the POB group, and the difference was statistically significant. Allelic richness ranged from 1.49 (H) to 1.95 (STH) with a mean of 1.78. The differences in the levels of this coefficient among the groups were statistically significant.

The analysis of the heterogeneity levels of accessions in the studied groups showed differences: DC (0.055–0.236), F (0.055–0.346), H (0.061–0.140), P (0.055–0.185), POB (0.058–0.235), and STH (0.055–0.365). In general, among the accessions tested, DC57/14 had the highest homogeneity, while STH_37 had the highest internal heterogeneity (Figure S1).

### 2.3. Genetic Relationship 

**Figure 3:**
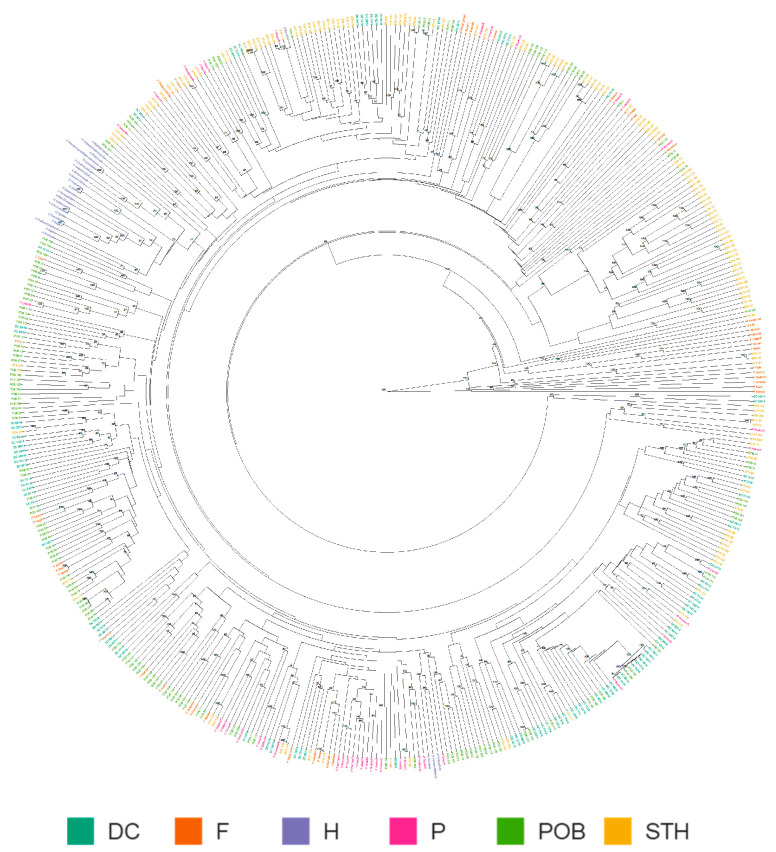
Hierarchical circular clustering dendrogram of 487 *A. sativa* genotypes based on 7411 SNPs generated using the UPGMA method and Provesti’s distance matrix with 1000 bootstraps. Different colors indicate different genotype groups: Historic cultivars (H), foreign cultivars (F), and breeding lines from the ongoing programs of breeding at Małopolska Plant Breeding Ltd. (POB), Strzelce Plant Breeding Ltd. (STH), and Danko Plant Breeding Ltd. (DC).

A dendrogram of 487 A. sativa cultivars and breeding lines based on Provesti’s distance grouped the analyzed genotypes into 15 clusters. Within the largest cluster, two subgroups were distinguished. The first subgroup was composed of 435 objects, all Polish (41) and historic cultivars (26), 30 out of 43 foreign cultivars, 134 out of 136 POB lines, 94 out of 96 DC lines, and 109 out of 145 STH lines (Figure 3). The second subgroup contained 33 STH lines and one POB oat-breeding line (POB 20). Two DC lines (DC64/14 and DC65/14) and one STH (STH 121) line built another cluster. The remaining foreign cultivars, four STH lines (STH 35, STH 42, STH 43, and STH 54), and the POB 122 line formed separate clusters. The most distant were Kanota, Quoll, Poto-roo, Wallaroo, Ugorny, Ogle, STH 42, STH 43, and STH 54.

**Figure 4:**
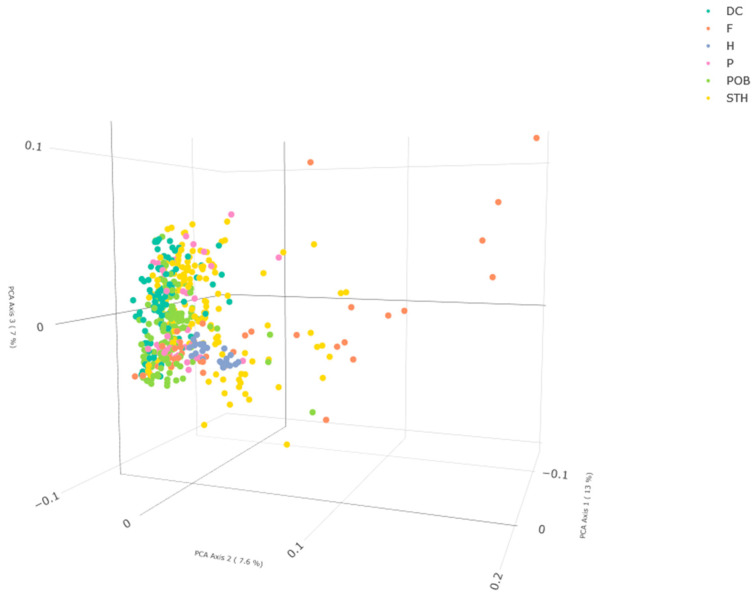
Three-dimensional plot of the principal coordinate analysis results for DArTseq data of 487 *A. sativa* accessions. Results in the first three coordinates’ system. Each point denotes one tested accession. RouTable 3D figure can be found in the Supplementary Materials (Figure S2). DC—materials from DANKO Plant Breeding Ltd.; F—foreign modern cultivars; H—historic cultivars; P—Polish modern cultivars; POB—materials from Małopolska Plant Breeding Ltd.; and STH—materials from Strzelce Plant Breeding Ltd.

To visualize the grouping pattern and provide a graphical representation of the relationships among cultivars, principal coordiate analysis (PCoA) was conducted. PCoA supplemented the above data with congruent results, which confirms the cluster analysis (Figure 4). The first three coordinates explained approximately 13%, 7.6%, and 7% of the total variance, respectively. The analysis showed that most of the examined cultivars and materials from the Polish breeding programs formed a central cluster in the 3D space. A significant part of the STH accessions, some foreign F cultivars, and a single POB accession stood out from this grouping. A detailed analysis of the groups made it possible to identify more subtle differences. Within the historical cultivars (H), two groups consisting of 12 and 13 accessions were clearly visible. It can also be seen that cultivar Borek was significantly separated from these two groups and located in the space occupied by the STH accessions. Contemporary Polish cultivars (P) formed a distinct cluster of about 25 accessions, which was directly adjacent to one of the clusters of historical cultivars. The remaining ones formed a loose cloud slightly away from the center of the analyzed space in the direction of accession ‘Swan_mut’, which was used in the breeding programs in which these cultivars were developed. Foreign cultivars were also largely located in the central part of the 3D space. A total of 13 cultivars were separated from this cluster and formed a very loose cloud that moved away from the central cluster along increasing PCA2 and PCA3 values. All of these cultivars were from breeding programs conducted outside of Europe. The POB and DC accessions occupied a very similar space to the Polish contemporary cultivars. The STH accessions showed the greatest diversity among the analyzed Polish breeding materials. Only a small group, i.e., about 15 accessions were located in the immediate vicinity of historical cultivars. The remaining accessions were significantly displaced from the central cluster, and this displacement ran in relation to PCA1 (in the descending direction) and PCA3 (in both directions). Thus, this cloud was separated from that formed by foreign cultivars (F). 

**Figure 5:**
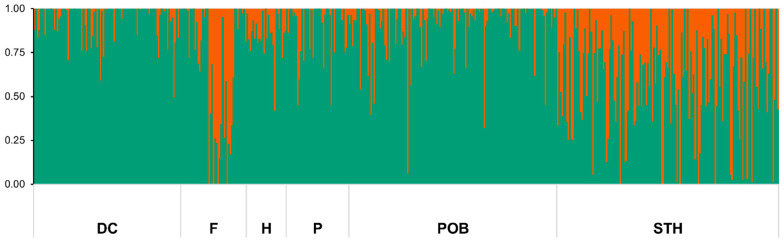
The results of genetic structure analysis using STRUCTURE 2.3.4 software [24] for 487 *A. sativa* accessions based on DArTseq-derived SNPs data with K = 2 based on ad hoc measure ∆K [25,26], where K is the number of ad hoc clusters; each vertical bar represents one accession. The length of the colored segment shows the estimated proportion of the membership of each gene pool in the cultivar genetic makeup. DC—materials from DANKO Plant Breeding Ltd.; F—foreign modern cultivars; H—historic cultivars; P—Polish modern cultivars; POB—materials from Małopolska Plant Breeding Ltd.; and STH—materials from Strzelce Plant Breeding Ltd.

The Bayesian model approach implemented in STRUCTURE 2.3.4 software was used for population structure analysis. When all the genotypes were analyzed, the ΔK peak was the highest for K = 2, supporting the presence of two distinct, major gene pools at a ratio of 80% to 20%. The ΔK value of 3791 indicates that these two detected pools had significant genetic distinctiveness. The first major gene pool had the largest presence in the DC, H, P, and POB genotype groups. The second major gene pool was the strongest in F and STH (Figure 5). Using the same admixture threshold as in an earlier study by Koroluk et al. [13], i.e., 0.8, about 30% of the accessions studied here represented admixed forms. 

To identify more subtle differences in the population structure of the studied breeding materials and cultivars, STRUCTURE analysis was performed for each group separately. The results show that two gene pools were also separated for the foreign cultivar (F) group (Figure S3). The first pool included 27, and the second pool included 4 cultivars, while 12 cultivars were considered to be admixed. In the overall population structure of this group, the first gene pool accounted for 75% and the second for 25%. It should be noted here that the second gene pool was associated with cultivars from breeding programs conducted outside of the European continent, i.e., in Australia and North America. 

A correlation analysis showed that the distribution in this group highly mirrored that described for the entire set of accessions studied here (0.979; *p* < 0.0001). Thus, the second major gene pool described in the genetic structure of the 487 accessions was related to sources of variation used in breeding programs conducted outside of Europe. The secondary structure of this group indicates the presence of as many as seven gene pools with the following proportions: 66%, 11%, 8%, 4%, 4%, 4%, and 3%. 

It is noteworthy that the cultivars bred in Europe still represented the first gene pool. The contribution of the other gene pools in these cultivars was small. Only the ratio of the fourth pool was in the range of 15–20% in cultivars bred in the United Kingdom and France. For Canadian cultivars, the second gene pool was typical, and for Australian cultivars, the third. It is evident that the flow of breeding material among programs on different continents was significantly lower than that among European countries. For US cultivars, the influence of European and Canadian components could be seen. 

In the group of Polish contemporary cultivars (P), the proportions of three gene pools were evident with 55%, 26%, and 18% (Figure S4). A total of 16 cultivars represented the first gene pool, three represented the second, and one represented the third. In addition, 51% of the cultivars contained two or three gene pools. Compared to the two major gene pools present in all accessions studied, the first gene pool was strongly correlated with the first major gene pool (0.645; *p* < 0.001), and the third was correlated with the second major gene pool (0.925; *p* < 0.0001). 

This indicates that the pedigrees of Polish contemporary cultivars included components from breeding programs conducted outside of Europe. This result is, therefore, consistent with the picture obtained from projecting the results of the first three main PCoA coordinates. The secondary genetic structure of this group indicates the presence of seven gene pools with the following proportions: 40%, 19%, 18%, 10%, 7%, 3%, and 3%. 

**Figure 6:**
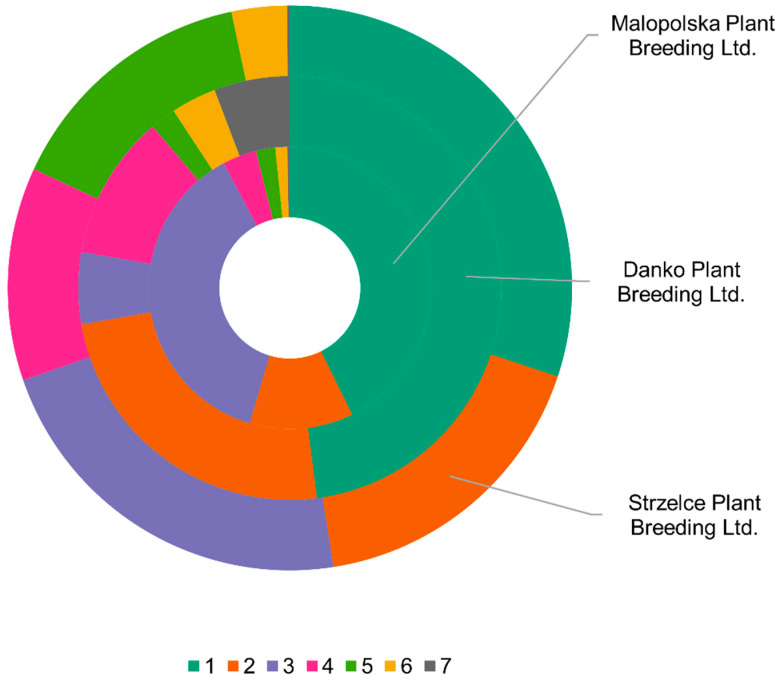
Proportions of seven gene pools in Polish modern cultivars depending on breeding company based on population structure analysis for *A. sativa* DArTseq SNPs.

Only five cultivars were classified as representing a single gene pool, and they all represented the first gene pool. The analysis of cultivars from the three Polish breeding companies showed differences in the percentages of individual gene pools (Figure 6). While the first gene pool was best represented in all three companies and made up 30% to 48%, only at Danko Plant Breeding Ltd. were all seven gene pools detected. At Malopolska Plant Breeding Ltd., the components used for crossbreeding represented the first three gene pools. On the other hand, Strzelce Plant Breeding Ltd. substantially used components from five gene pools. 

The primary genetic structure of the historical cultivars (H) showed the presence of three gene pools whose shares were 43%, 29%, and 28% (Figure S5). Five cultivars were assigned to the first, four to the second, and two to the third gene pool. The remaining 15 cultivars showed mixed ancestry. Pools 2 and 3 only moderately corresponded with the major pools 1 and 2 determined for all accessions (0.530; *p* = 0.005 and 0.573; *p* = 0.002, respectively). The second-order structure determined for k = 6 showed that there was no change in the pre-determined first and second gene pools. The four new gene pools were actually derived only from the third gene pool (11%, 10%, 5%, and 5%). One cultivar was assigned to each of the three gene pools, and the fifth gene pool was only an admixture.

For the DC breeding lines, the primary structure was also formed by two gene pools (Figure S6). Of the lines, 42.7% were classified into the first and 11.5% into the second gene pool, and the remaining 45.8% were admixed forms. Although the first-order structure is quite strong, it should be kept in mind that the two gene pools distinguished in this group did not correspond to the major pools determined during the analysis of 487 accessions. This result was confirmed by the correlation analysis (0.192; *p* = 0.061). The analysis also showed the presence of a secondary population structure for k = 3; however, the probabilities of its occurrence were significantly lower. After separating the three gene pools, only 38 accessions were considered pure (25, 11, and 2 for gene pools 1, 2, and 3, respectively), and as many as 58 accessions, i.e., 60% were admixed. The proportions of individual gene pools in the studied materials were 56%, 31%, and 13%. However, the correlation analysis showed that the third segregated gene pool largely corresponded to the second major gene pool. 

For the STH group of breeding materials, we also found that two major gene pools most likely formed its diversity and genetic structure (Figure S7). In this case, the correlation analysis showed that the two identified pools of the first-order structure significantly corresponded to the major pools separated in the analysis of all accessions (0.828; *p* < 0.0001). Of the 145 accessions examined, as many as 74% were assigned to the first gene pool, and only 9% to the second one. In contrast, 17% of the accessions were considered to be admixed. Due to the very strong first-order structure (ΔK = 4237.6), the probability of a lower-order structure in this group was quite low, i.e., ΔK = 22.9 for k5. With such parameters applied, only 23 accessions were classified as representing the first gene pool. Six accessions each represented the second and third gene pools, and only two for the fifth gene pool. The fourth gene pool occurred only as an admixture, and its contribution to the studied group of accessions was about 9%. The participation values of the other gene pools in this group of breeding materials were 41%, 30%, 13%, and 6% for the first, second, third, and fifth gene pools, respectively.

Regarding the third group of contemporary Polish breeding materials, i.e., POB, the algorithm implemented in the STRUCTURE 2.3.4 software showed that three gene pools were most likely to be involved in this group (Figure S8). Totals of 69, 9, and 3 accessions were assigned to these three gene pools, while 55, i.e., 40.4%, were considered to be admixed. The contributions of the three pools to the overall genetic makeups of the groups were 69%, 20%, and 11%, respectively. The correlation analysis showed a significant relationship between this structure and the two major pools determined in the analysis of all accessions. 

The third POB gene pool significantly reflected the second major gene pool (0.976; *p* < 0.0001), and the other two POB gene pools were contained within the first major gene pool determined within 487 accessions. A secondary structure in this group singling out four gene pools was highly probable. There were 32, 9, 7, and 3 accessions classified as pure forms to the respective gene pools, and as many as 62.5% of the accessions were considered to be admixed in this case. Here, pools 1 and 2 were separated from the first gene pool in the primary structure of this group, and pools 3 and 4 almost completely corresponded to the previously described pools 2 and 3. 

### 2.4. Lost and Gained Alleles

An assessment of the proportions of unique loci in the studied groups was performed. The analysis was performed according to the method of Dziurdziak et al. (2022), i.e., only loci that were quite common in one of the groups were considered (i.e., only loci with a frequency higher than 0.25, and at the same time, in the compared second group with a frequency lower than 0.05). This analysis showed that the individual groups differed significantly in terms of unique alleles. The smallest differences occurred when comparing Polish modern cultivars and POB breeding materials from the Małopolska Plant Breeding Ltd. 

**Figure 7:**
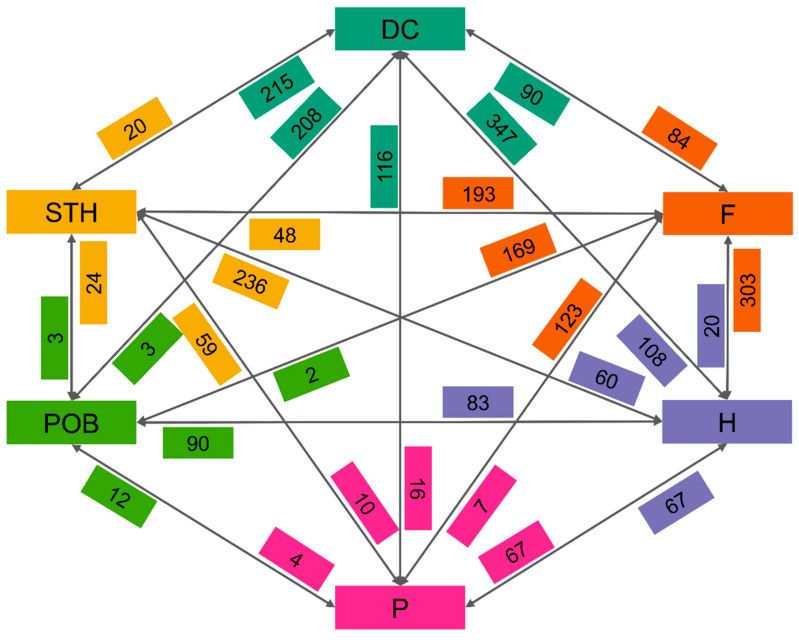
Summary of changes in the number of unique alleles during more than 120 years of breeding and cultivation of oats in Poland. Colors indicate groups, and arrows connect compared periods. DC—materials from DANKO Plant Breeding Ltd.; F—foreign modern cultivars; H—historic cultivars; P—Polish modern cultivars; POB—materials from Małopolska Plant Breeding Ltd.; and STH—materials from Strzelce Plant Breeding Ltd.

The two groups differed by only 16 alleles, which indicates a lack of inflow of new alleles into the gene pool used by the breeders working there and a focus on handling the genetic variability present in the domestic market (Figure 7). The other two breeding companies showed a higher number of new alleles absent in contemporary Polish cultivars, i.e., 59 for STH and 116 for DC. On the other hand, there were between 84 and 169 alleles in the foreign cultivars that were not present in contemporary Polish breeding materials. At the same time, the gene pool of the DC materials contained 90 alleles absent in the foreign cultivars. For STH, this amounted to as few as 48 alleles, and POB had only two alleles that were unique compared to the foreign cultivars. 

Comparing historical cultivars and modern Polish cultivars, it can be seen that, during the breeding process of over 100 years, 67 alleles from the old gene pool were lost and replaced by 67 new alleles. However, it is interesting to note that, while as many as 303 alleles present in the modern foreign cultivars were not present in the historical cultivars, only 20 alleles unique to the historical cultivars were not retained in the foreign cultivars. Current breeding programs have introduced between 90 (POB) and 347 (DC) new alleles into the gene pool relative to cultivars from the early days of breeding. In contrast, there were 12 (POB) to 116 (DC) new alleles compared to the modern cultivars. At the same time, the modern breeding materials did not contain 60 (STH) to 108 (DC) alleles present in the historical cultivars. 

### 2.5. Swan Mutant Case in Polish Breeding

**Figure 8:**
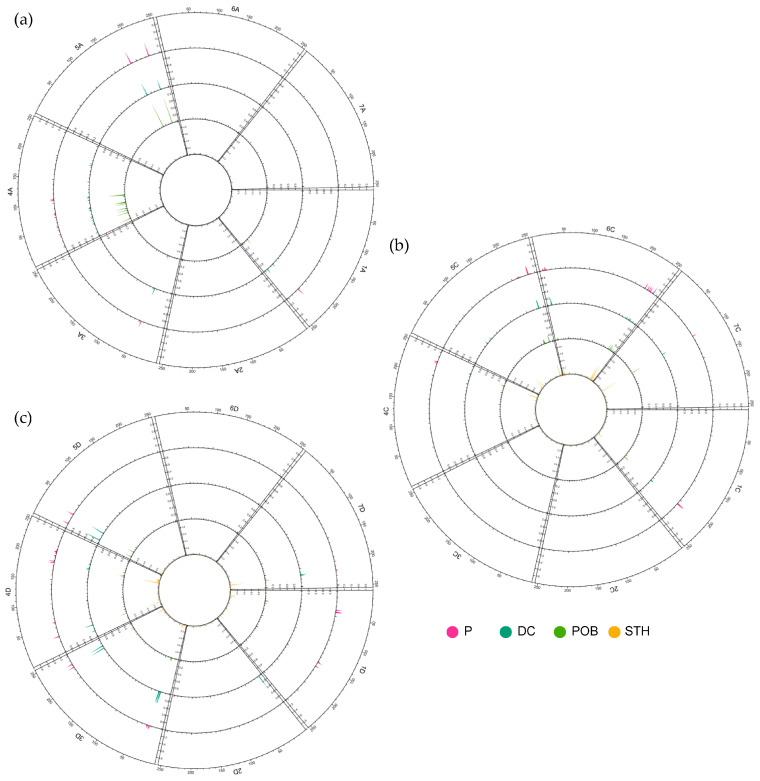
Loci containing alleles unique to Swan mut present in Polish *A. sativa* cultivars and breeding materials; (**a**) A sub-genome; (**b**) C sub-genome; and (**c**) D sub-genome. DC—materials from DANKO Plant Breeding Ltd.; P—Polish modern cultivars; POB—materials from Małopolska Plant Breeding Ltd.; and STH—materials from Strzelce Plant Breeding Ltd.

The pedigree analysis of Polish modern varieties showed that, in breeding programs, Swan mut appears quite often as one of the components. A total of 142 alleles unique to Swan mut accessions were identified. Loci containing these alleles were present in all three homoeologous sub-genomes; however, their abundances varied (Figure 8). The largest number of unique loci was detected in the D sub-genome (40.8%). Furthermore, 25.4% were in the C sub-genome, and 18.3% were in the A sub-genome. For 15.5% of the unique loci, the chromosomal location was unknown. 

From a chromosome perspective, the largest number of loci was found on chromosome 4D (34 loci). Four chromosomes, i.e., 2A, 3C, 6A, and 6D, did not carry any loci containing a unique allele. The analysis showed that, within Polish breeding materials and cultivars, all these alleles occur; however, their numbers and frequencies varied. The highest number of unique alleles was found in STH breeding materials, and the lowest in POB. The mean frequency of unique alleles was also the highest in STH and significantly different (*p* < 0.0001) from the mean frequencies in P (0.084) and POB (0.075). The distribution of unique alleles along the chromosomes showed that they occur in clusters.

### 2.6. Targeted Selection

**Figure 9:**
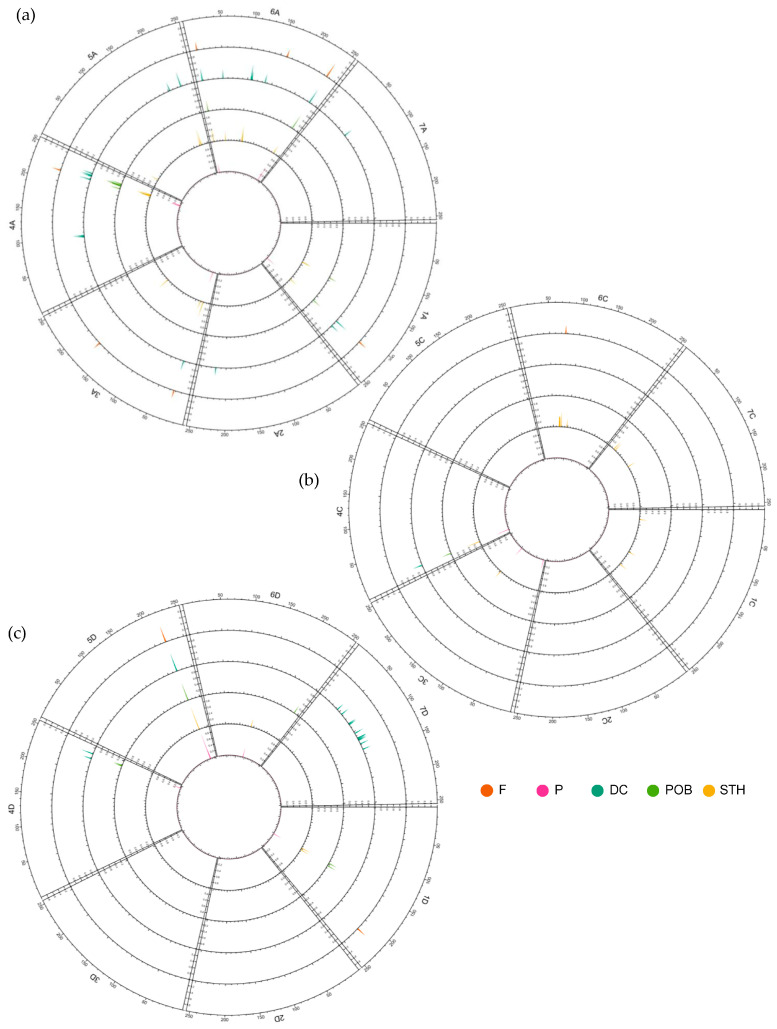
Circular overview of A. sativa chromosomes with an indication of the FST values for historic cultivars vs. other groups. (**a**) A genome chromosomes, (**b**) C genome chromosomes, (**c**) D genome chromosomes. DC—materials from DANKO Plant Breeding Ltd.; F—foreign modern cultivars; P—Polish modern cultivars; POB—materials from Małopolska Plant Breeding Ltd.; and STH—materials from Strzelce Plant Breeding Ltd.

To determine the region in which breeding-related changes have occurred from the origins in Poland to the present day, a pairwise F_ST_ coefficient analysis was performed for the historical cultivar group as well as for all contemporary groups of breeding materials and cultivars (Figure 9). This analysis showed that, relative to the historical cultivars, most changes occurred in the A sub-genome, especially on chromosome 6A. Significantly, the regions where changes occurred were distributed along the entire chromosome—not only at the ends. 

Breeding programs have made almost no changes to chromosomes 2A and 7A. Only the DC materials showed one region each on these two chromosomes where there was a clear change in the allele frequency. At the ends of chromosomes 1A and 4A, there were regions where all the tested groups showed significant, targeted changes from the historical cultivars. On the other hand, in the case of 3A, the allele frequency did not differ significantly between the POB group and the historical cultivars. 

In the C sub-genome, targeted changes were definitely the rarest. All groups of domestic breeding materials showed the presence of changes at the end of chromosome 4C. Interestingly, this change was not observed in the group of modern Polish cultivars. The STH materials showed the most changes within this sub-genome in relation to the historical cultivars. In the group of foreign cultivars, there were virtually no regions where directed selection occurred relative to the historical cultivars. 

In the D sub-genome, a clearly targeted change occurred at the end of chromosome 5D. In contrast, on chromosomes 2D and 3D, breeding programs lasting more than 100 years did not introduce any significant changes. In the group of DC materials bred by Danko, the occurrence of very significant changes in the allele frequency was observed, and interestingly, these changes tended to affect the central part of the chromosome. On chromosome 1D, it was observed that materials from the Polish contemporary breeding programs showed a different region of directed changes than the foreign cultivars.

### 2.7. Core Collection

The minimum group of cultivars representing the full diversity was identified using an advanced maximization strategy through a modified heuristic algorithm (A*), which is complete and optimal. Out of the studied 487 accessions, a set of 289 that should form the core collection was extracted. The core collection included 27 Polish modern cultivars, 35 foreign cultivars, 15 historical cultivars, 54 DC, 81 POB, and 76 STH materials. The cultivars are marked in Table S1.

## 3. Discussion

In this study, DArTseq technology was applied to evaluate 487 oat accessions, including breeding lines from the ongoing programs of the three Polish breeding companies along with modern and historical Polish and foreign cultivars. Thus far, in the *Avena* studies, DArTseq has been successfully used for molecular marker identification, mainly for the detection of markers linked to disease resistance [27,28,29]. In oat, as in other polyploid species, due to the presence of homoeologous sub-genomes, genotyping is complicated and requires marker filtering to eliminate those that are confounded by multiple loci [30]. 

In the analyzed panel, we selected 32,253 markers comprising 7411 high-quality, non-redundant SNPs. The markers were distributed among three sub-genomes (A, C, and D) with the greatest number of markers in sub-genome D (2646). An uneven distribution of markers among sub-genomes has been previously reported, e.g., in wheat [18]; however, in our study, the observed disproportions were much lower. Some wheat studies have indicated that the distribution of identified SNPs may be influenced by differences in the sub-genomes because a smaller proportion of markers was mapped to the “youngest” sub-genome D compared to sub-genomes A and B [31,32,33,34].

In our study, we observed a low or complete absence of SNPs in the centromeric and pericentromeric regions and high frequency in the distal parts of chromosomes. The DArTseq library was based on the *PstI/TaqI* genome complexity reduction similar to the DArT microarray [35,36]. Other marker technologies based on methylation-sensitive restriction enzymes have shown the same predisposition of clustering in various regions with little or no coverage in other regions [37,38]. Marker distribution may reflect the overall protein-coding gene dispersal. This has been observed in studies on barley [20,39], wheat [40,41], and, to a lesser extent, rice [42,43]. In these species, the levels of recombination and marker frequency were suppressed near the centromere and increased in telomeric, gene-rich, euchromatic chromosome regions.

In the studied *Avena* set, all possible SNP types were detected with slightly more transition-type (53.05%) than transversion-type (46.95%) mutations. The excess of transition SNPs has also been observed in previous studies, e.g., for barley, wheat, rice, cowpea, and common bean [44,45,46,47,48], and has occurred due to the higher probability of preserving protein function and structure [47,49]. 

Overall, the most frequent nucleotide change was A > G, followed by C > T, which is consistent with the above-cited research and may be connected with the frequency of methylation/demethylation-related mutations. However, in the D sub-genome, the most abundant SNP was G > A. In the case of transversions, the most abundant SNP was C > G, except for the C sub-genome, where the most frequent change was G > C—yet, its frequency was comparable to C > G. Similar results have been obtained by DArTseq and GBS for barley [20,45] and wheat [45].

The heterozygosity in the studied *Avena* population was very low with a mean value of 0.10. Similar values, typical for self-pollinated species, were obtained in the genetic structure study of the 509 wheat accessions representing registered varieties and advanced breeding lines using the GBS approach [18]. Advanced breeding materials should be highly homozygotic, so as expected, the minimum Ho value was observed in the group of Polish modern cultivars (P). This confirms the genetic uniformity of individuals within a cultivar resulting from the breeding process. 

The analyzed loci were localized on the reference *A. sativa* genome, allowing for tracking if changes in the genetic diversity were related to the sub-genome or individual chromosomes. In general, the proportion of heterozygous loci in each sub-genome was relatively equal. At the chromosome level, heterogeneity was dependent on the analyzed group and was the highest on 5A and 6A and the lowest on 7C and 4A, which represented regions of low variation. It should be noted that, for virtually every chromosome, there was a group of outlier loci that had a very high level of heterogeneity.

In previous research conducted on large panels of oat lines based on DArT or GBS markers, a weak population structure within the analyzed accessions has been observed [30,50,51,52,53,54,55,56,57,58]. Authors have claimed that oat, despite having four recognizable types (naked, covered, spring, and winter), has not shown an obvious structure of a population. In our material set, the majority of accessions were covered-seeded spring oat, as Polish breeding is based on this type of line due to the climatic conditions of the country and market requirements. 

Naked or winter lines were added to the evaluated set within foreign cultivars, and both dendrogram and PCoA analysis showed that some foreign cultivars stood out from the grouping. Additionally, these analyses indicate a significant part of Strzelce Plant Breeding lines (STH) and single Małopolska Plant Breeding lines (POB) as distinct. Similarly, the STRUCTURE analysis showed that STH accessions had the greatest diversity among the analyzed Polish breeding materials. The results also indicate that the pedigrees of Polish contemporary cultivars included components from breeding programs conducted outside of Europe, which indicates the thriving development of Polish breeding and confirms the systematic inflow of breeding materials. 

However, the data show that the exchange of breeding materials among programs on different continents was significantly lower than that among European countries. In the past, high yield was the main direction of oat breeding due to its main usage in livestock feeding. Currently, many studies have confirmed a wide range of possible oat utilization in the food, pharmaceutical, cosmetic, and chemical industries, and hence the new oat breeding directions may be reflected in the resulting groups within the analyzed breeding lines when the analysis is performed for each group separately.

Modern oat breeding is based on constant selection from the progeny of crosses among related, current, or recent cultivars. With time, heterogeneous landraces have been replaced by varieties that are mostly homogeneous populations [14]. This allowed for obtaining high yield and better adaptation to farming conditions; however, the risk of a significant narrowing of the breeding materials’ gene pools emerged. Purposeful breeding activities may cause ‘genetic erosion’ [59], manifesting in a dramatic shift in the population structure or allele frequencies within a species, as exemplified in wheat by the Green Revolution of the 1960s and 1970s [60]. In our study, the obtained results indicate that no erosion of genetic diversity was observed within the Polish native oat gene pool. 

During the 120 year oat-breeding process, only 67 alleles from the old gene pool were lost and replaced by 67 new alleles, whereas, in Polish spring barley, the losses concern about 600 alleles that are preserved only due to the activity of the gene bank [20]. Interestingly, a significant part of the variability of old oat varieties has been preserved in foreign cultivars. It is worth noting that the current oat breeding programs have introduced 673 new alleles into the gene pool relative to historical cultivars. In contrast, there were only 187 new alleles in the contemporary breeding materials compared to the modern cultivars. 

A detailed analysis of changes in the allele frequency showed that POB breeding is based on local contemporary materials, and limited cross-breeding with new sources of variation is manifested in the absence of alleles unique to Polish modern cultivars. Although PCoA and STRUCTURE showed significant differentiation in the STH materials, there were fewer unique alleles in relation to the modern varieties in comparison to DC. Most likely, STH started introducing foreign sources of variability earlier than DC, and therefore modern varieties already contain new variability. The allelic richness and large dispersion of the STH genotypes on the PCoA chart may indicate that the STH breeding process also uses new sources of diversity, and new alleles are not yet fixed in the population of breeding materials. 

The pedigree analysis of Polish modern varieties, especially those derived from the Strzelce Plant breeding station, showed that Swan mut was often used for crossing. Swan is an Australian dual-purpose (hay and grain) spring cultivar that was released in 1967 and derived from a cross between Kent (USA) and Ballidu (Western Australia) [61]. It is characterized as being very tall and having medium maturity, high quality, plump grain, a high groat content, excellent hectoliter weight, good digestibility, and a lower protein content in hay. 

In Polish breeding, the single plant selected from the Swan cultivar called ‘mutant’ with very promising panicle architecture parameters and thousand-grain weight, was used for crossing at the Strzelce breeding company (Z. Nita, personal communication). The first Polish cultivar with Swan mut in its pedigree, which was registered in 1993 and withdrawn from the register 27 years later, was Sławko [62]. This cultivar was highly appreciated by the farmers for its stress tolerance [63] and resilience to environmental changes. Swan mut appeared in the pedigrees of numerous Polish cultivars released after 2000, for example, among others, in the best Polish cultivar Bingo, which is well-known in the European market. 

In our studies, 142 alleles unique to Swan mut were identified with the largest number detected in the D sub-genome (40.8%), out of which the largest number of loci (34) was located on chromosome 4D. The distribution of unique alleles along the chromosomes showed that they occur in clusters. De Koeyer et al. [64] and Tinker et al. [65] localized QTLs on chromosome 4D that are responsible for the grain yield, 1000 kernel weight, test weight, lodging severity, plumpness of kernels, and milling yield. On the 4D chromosome, QTLs connected with the flowering time, photoperiod, and vernalization response have also been found [66,67]. All these features are agronomically important, and novel alleles that condition more favorable trait values could be subjected to targeted selection in the breeding process.

F_ST_ analysis enabled the identification of regions in which breeding-related changes have occurred. Different alleles were fixed in the contemporary breeding materials, and cultivars were compared to the obsolete cultivars. This analysis revealed that, relative to the historical cultivars, targeted changes were the rarest in the C sub-genome. Oat genomes, as a consequence of frequent genomic rearrangements, have resulted from a mosaic-like architecture [68,69]. As a result of translocation events in oat, the sub-genomes exhibit unbalanced gene numbers. An examination of the oat chromosomes revealed the lowest gene counts in the C sub-genome as a consequence of the translocations of gene-rich regions from the C to the A and D sub-genomes [70,71]. Examples of the lack of genes on the C sub-genome are grain proteins, avenins, and globulins—encoding genes identified only on the chromosomes of sub-genomes A (1A, 4A, and 7A) and D (1D, 3D, and 4D) [70]. 

Intriguingly, the assembly results show that due to the abundance of repetitive sequences, sub-genome C is about 20% larger than A and D [71]. Moreover, the study of sub-genomic gene expression differences revealed the bias in homoeologous gene usage and pointed out the C sub-genome genes as slightly less expressed than those derived from the D or A sub-genomes [70,71]. An examination of the non-synonymous-to-synonymous substitution rate ratios showed that the purifying selection of the C sub-genome was less intense than that of the other two sub-genomes [71]. Nevertheless, some QTLs for agronomically important traits were identified on sub-genome C, such as the oil content (4C), husk percentage (6C), and loci related to local adaptation (1C and 3C) [65,67].

The most changes within oat genomes in relation to the historical cultivars occurred in the A sub-genome with emphasis on chromosome 6A, where changes are distributed along the entire chromosome—not only at the ends. Chromosome 6A contains major QTLs and candidate genes for oil content (ACC.416), β-gulcan (CslF9 and CslF11), and the 1000 kernel weight [65,72,73,74,75]. Our studies revealed that breeding programs lasting more than 100 years have made almost no changes on chromosomes 2A and 7A, despite previous surveys locating on these chromosomes the loci influencing the groat percentage (2A) and flowering time (7A) [65,76]. 

A clear change in the allele frequency on these two chromosomes was visible only in the DC materials. All the tested groups showed significant targeted changes in comparison to the historical cultivars at the ends of chromosomes 1A and 4A. At the end of chromosome 1A, the QTLs for the groat percentage and milling yield were identified, whereas, at the end of 4A, major loci related to the heading time, plant height, and lodging severity were found [65,77,78]. A clearly targeted change occurred at the end of chromosome 5D, on which QTLs for the oil content in grain were previously discovered [75]. 

Contrarily, on chromosomes 2D and 3D, the breeding programs did not introduce any significant changes. On the 3D chromosome, no QTL was found in previous studies; however, on the 2D chromosome, loci affecting the 1000 kernel weight, lodging severity, and groat percentage were established [65]. The identification of regions subjected to targeted selection revealed that Polish oat breeding goals were typical for this crop and focused on enhancing the grain yield and quality and improving adaptability. 

In our study, using the high-density SNP genotyping data, we identified a core set of cultivars and lines, thereby, capturing the majority of the allelic diversity present in the Polish breeding materials and cultivars. The core collection included a group of 289 cultivars and lines representing the total genomic variation. This set can be used for evaluating traits that are difficult or expensive to score. Particular emphasis should be placed on maintaining these breeding materials and cultivars as germplasm collections in gene banks to facilitate the future use of these genetic resources by scientists and breeders.

## 4. Materials and Methods

### 4.1. Plant Material

The plant material consisted of 487 *A. sativa* accessions (Table S1), of which 41 were Polish modern cultivars (P), 26 were historic cultivars (H), and 43 were foreign cultivars (F) from the collection of the Institute of Genetics, Plant Breeding and Biotechnology, University of Life Sciences in Lublin, Poland and the National Centre for Plant Genetic Resources, Radzików, Poland. The remaining genotypes were breeding lines from the ongoing programs of the largest Polish breeding companies—Małopolska Plant Breeding Ltd. in Polanowice (136 genotypes designated as POB), Strzelce Plant Breeding Ltd. (145 genotypes designated as STH), and Danko Plant Breeding Ltd. (96 genotypes designated as DC).

### 4.2. DNA Extraction and Genotyping

Extraction of the total genomic DNA from the analyzed forms was conducted from the young leaves of 10 seedlings obtained from seeds taken from a single random plant using the DNasyTM Plant Mini Kit (Qiagen^®^, Hilden, Germany) following the manufacturer’s protocol. The quality and quantity of extracted DNA were evaluated spectrophotometrically (NanoDrop2000). An analysis of the DNA’s integrity was performed by gel electrophoresis. The concentration of all DNA samples was adjusted to 100 ng µL^–1^.

DArTseq^TM^ analysis was performed by Diversity Arrays Technology Pty Ltd. (DArT P/L, Canberra, Australia), combining the DArT technique with next-generation sequencing [79]. DNA libraries were generated using genomic complexity reduction technology [16] by the digestion of the DNA samples with PstI and TaqI restriction enzymes (NEB) and ligation with adaptors corresponding to the enzyme overhangs. Only PstI-TaqI fragments were effectively PCR-amplified, followed by sequencing on an Illumina Hiseq 2500. 

Sequencing data were processed using proprietary DArT analytical pipelines, providing two types of markers: silicoDArT presence/absence variants (PAVs) analogous to microarray DArTs but extracted in silico from sequences obtained from genomic representations, and DArTseq single-nucleotide polymorphisms (SNPs) in fragments present in the representation. The sequences were then BLASTed against a reference genome, *Avena sativa* OT3098 v2, PepsiCo [23] with an expected value (E) < 10^−10^ and a minimum base identity of >95% as blast criteria.

### 4.3. Data Mining and Analysis

Population structure and diversity analysis were performed by implementing R-4.1.2 [80] in Rstudio (1.4.1106). As a first step, VCF files resulting from the marker calling were subjected to the filtering process by the dartR package [81]. Markers were removed if they were monomorphic, missing ≥5% of calls, had reproducibility (RepAvg) <1, or if the relative minor allele frequency (MAF) was <0.01. The matrix was transformed into genind, genpop, genlight, and hierfstat objects and subjected to further analysis in the R packages.

In the next step, the proportion of each mutation type was determined, and basic coefficients, such as the polymorphic information content (PIC), observed (Ho) and expected (He) heterozygosity, and inbreeding factor (F), were calculated [44]. A sliding window method with 500 kb intervals was used to assess the allocation of SNPs, PIC, and Ho along chromosomes in the three homoeologous sub-genomes. The results were presented as a circular layout as average values for 250 spots per chromosome using the circlize package [82]. The rarefaction method implemented in HP-RARE 1.0 was used to estimate the level of allelic richness (AR) [83]. Wright’s F_ST_ parameter was used to estimate the genome-wide diversity [84]. Analysis of variance (ANOVA) and Tukey’s post hoc test were used to verify the significance of the differences.

To assess the hierarchical levels of population structuring, an analysis of molecular variance (AMOVA) was computed among a priori assigned and novel clusters. Clustering was then performed based on the Bayes model for genetic structure analysis implemented in STRUCTURE 2.3.4 software (Pritchard Lab, Stanford University) [24]. To obtain the most probable value of K, a search was performed in the range of 1 to 20 with five independent repetitions per K and within groups of 1–10 with five repetitions per K. For each run, the initial burn-in period was set to 50,000 with 100,000 MCMC (Markov chain Monte Carlo) iterations after burn-in. 

For batch file analysis, a LINUX cluster hosted by the Interdisciplinary Center for Mathematical and Computer Modeling at the University of Warsaw was used. The number of clusters was determined by the *posteriori* data probability for a given K and ΔK [25], and a full search algorithm of CLUMPAK [26] was used to find the best match of the replicated cluster analysis results. A baseline value of 0.8 was defined as the probability of assigning accessions to the gene pool [13].

An advanced M strategy implemented through a modified heuristic algorithm (A*) was used to extract the core collection [85].

## 5. Conclusions

Precise genotyping is currently the first step to obtain marker-assisted breeding progress and to increase the efficiency of traditional breeding in crops. Therefore, to achieve genetic gain, it is important to have knowledge regarding the allelic diversity in the available genetic resources. Utilizing modern genomic tools enabled the detailed characterization of 487 cultivars and lines representing over 100 years of Polish breeders’ efforts. The performed analyses demonstrated that traces of directed selection are particularly visible on sub-genomes A and D—mainly on chromosome 6A and to a lesser extent on 5D. Notably, targeted changes were the rarest in the C sub-genome. 

This study shows that, in over 100 years of breeding in Poland, some alleles have been replaced by new ones; however, only a small number have been lost, and the Polish gene pool has avoided genetic erosion. The study also proves that Polish oat breeding based mainly on traditional breeding methods and focused on improving agronomically important traits, i.e., the grain yield and quality and environmental resilience and adaptability, did not significantly narrow the oat gene pool. 

Polish breeders have developed cultivars that are not only competitive in the European market but that also constitute a reservoir of new alleles that were not found in the analyzed foreign materials. Since we performed a comparison of the materials from three independent breeding programs, we can conclude that each of them has developed different breeding materials with different properties. This obtained knowledge is a pre-requisite for further association mapping studies of important agronomic traits.

## Figures and Tables

**Table 1:** Summary of point mutation abundance at the tested loci by chromosome based on DArTseq analysis of 487 *A. sativa* accessions.

		Transitions				Transversion								% Ts	% Tv	Ts/Tv Ratio
		Purines		Pyrimidines		Purines > Pyrimidines				Pyrimidines > Purines						
		A > G	G > A	C > T	T > C	A > C	A > T	G > C	G > T	C > A	C > G	T > A	T > G			
Abundance on chromosome	1A	39	40	35	51	20	7	27	14	11	38	6	14	0.546	0.454	1.204
	1C	23	26	27	13	10	3	17	10	8	20	5	4	0.536	0.464	1.156
	1D	47	74	55	70	18	14	49	29	37	48	13	16	0.523	0.477	1.098
	2A	17	23	24	16	7	5	20	9	7	10	3	7	0.541	0.459	1.176
	2C	60	50	47	44	24	6	18	16	16	29	10	28	0.578	0.422	1.367
	2D	49	64	57	47	12	11	34	24	23	34	5	25	0.564	0.436	1.292
	3A	32	23	20	25	8	3	17	9	15	11	2	7	0.581	0.419	1.389
	3C	39	43	27	26	18	8	32	17	17	23	2	14	0.508	0.492	1.031
	3D	27	32	27	24	13	3	23	13	14	25	4	12	0.507	0.493	1.028
	4A	53	40	35	43	13	10	34	25	16	33	9	18	0.520	0.480	1.082
	4C	59	47	49	49	24	6	37	11	29	35	9	24	0.538	0.462	1.166
	4D	67	68	71	66	22	16	52	37	38	50	15	22	0.519	0.481	1.079
	5A	22	23	18	28	9	6	11	12	8	21	4	14	0.517	0.483	1.071
	5C	46	41	49	42	22	6	30	17	18	28	13	10	0.553	0.447	1.236
	5D	57	59	52	34	19	8	41	29	21	46	14	24	0.500	0.500	1.000
	6A	33	20	30	28	14	9	13	14	13	29	5	10	0.509	0.491	1.037
	6C	38	37	42	55	15	7	34	21	15	31	10	12	0.543	0.457	1.186
	6D	17	29	22	16	4	1	10	10	15	16	6	7	0.549	0.451	1.217
	7A	36	36	43	34	13	2	26	14	12	26	8	23	0.546	0.454	1.202
	7C	39	38	33	32	14	3	23	21	18	15	6	13	0.557	0.443	1.257
	7D	66	65	62	60	32	12	46	23	36	44	17	30	0.513	0.487	1.054
Unknown		161	127	138	133	57	32	107	63	64	115	34	63	0.511	0.489	1.045

## Data Availability

Not applicable.

## Supplementary Materials

The following supporting information can be downloaded at: https://www.mdpi.com/article/10.3390/ijms24076547/s1.
