# Supplementary figures and images for: Utilizing Genomics to Characterize the Common Oat Gene Pool—The Story of More Than a Century of Polish Breeding
Source: Int J Mol Sci. 2023 Mar 31;24(7):6547. doi: 10.3390/ijms24076547 (PMC10094864; doi:10.3390/ijms24076547)

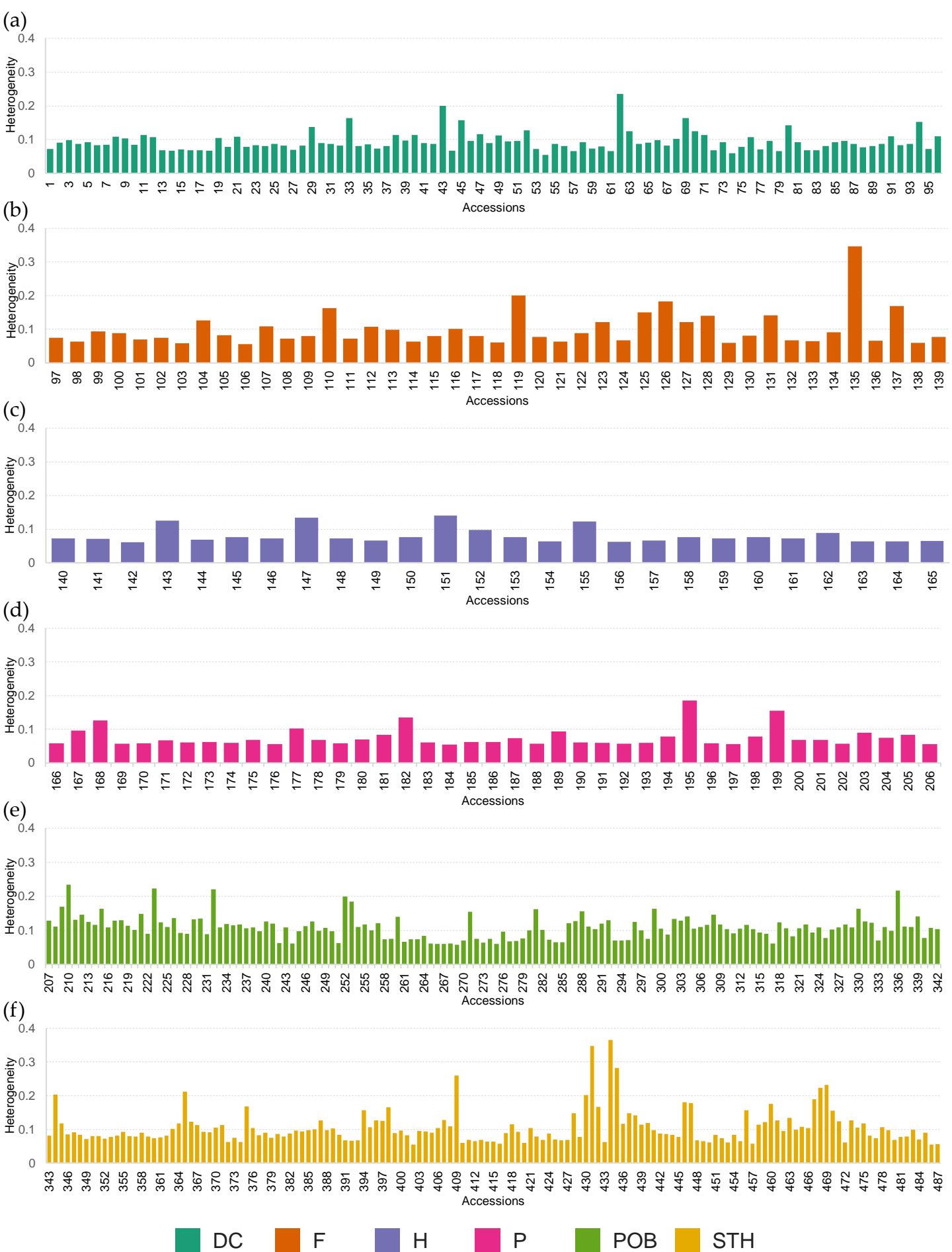

Supplement: Supplementary file 1 [file ijms-24-06547-s001.zip › Fig S1.pdf]

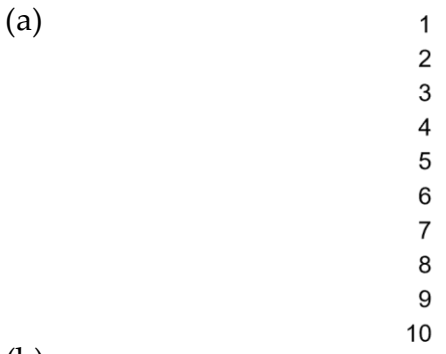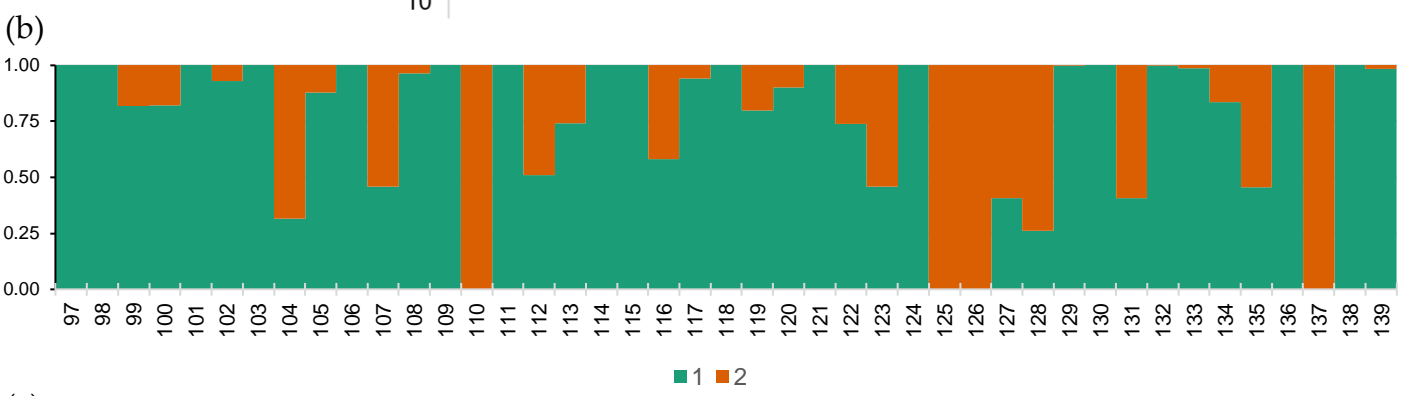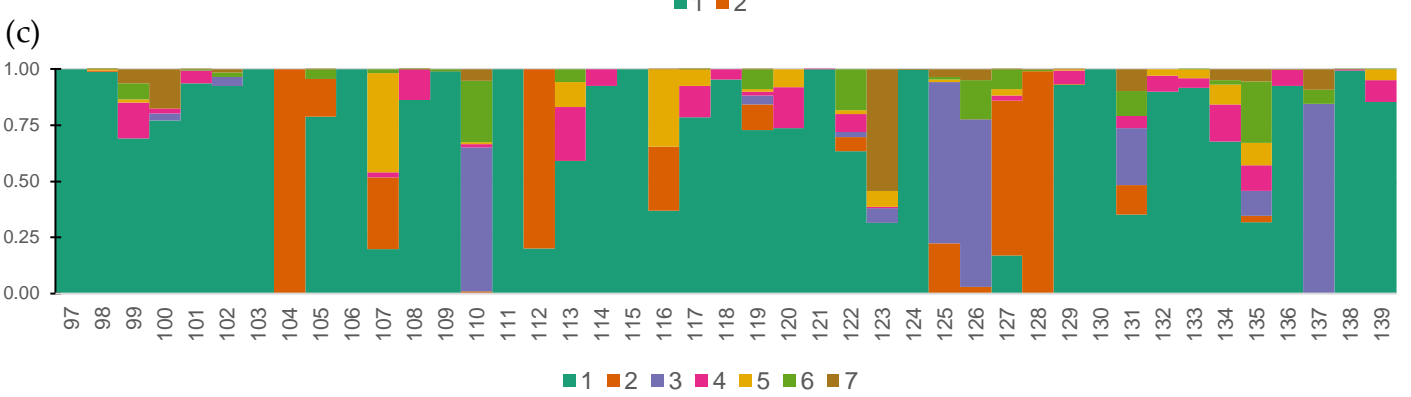

Supplement: Supplementary file 1 [file ijms-24-06547-s001.zip › Fig S3.pdf]

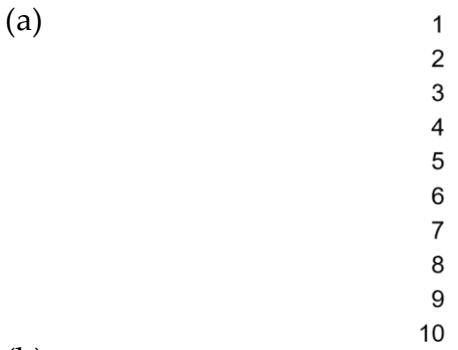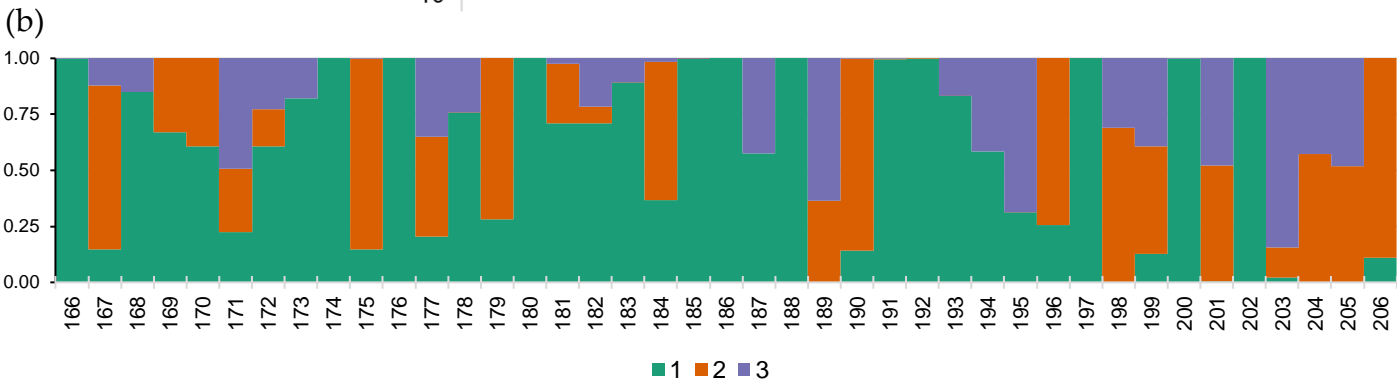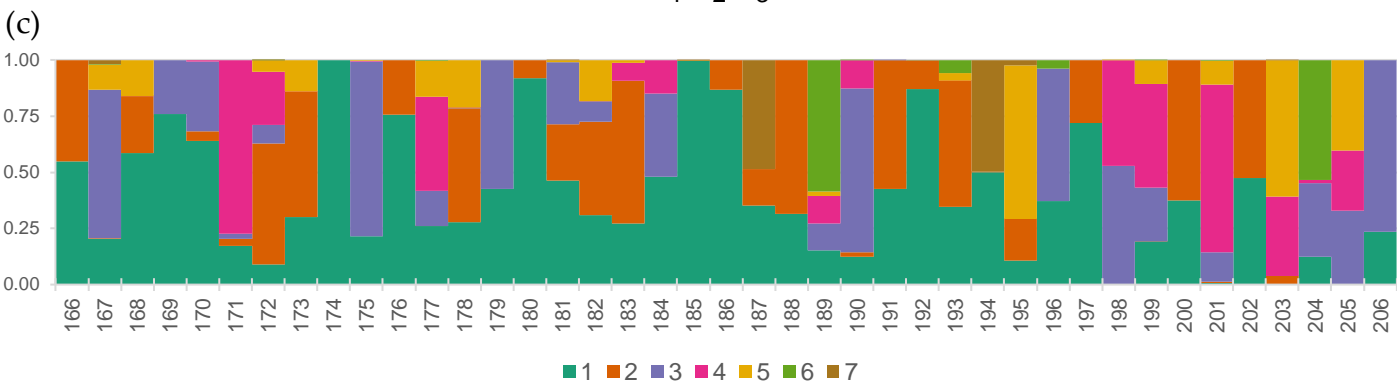

Supplement: Supplementary file 1 [file ijms-24-06547-s001.zip › Fig S4.pdf]

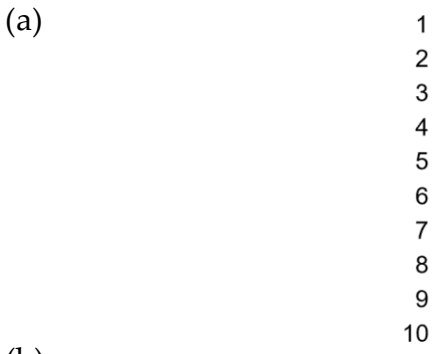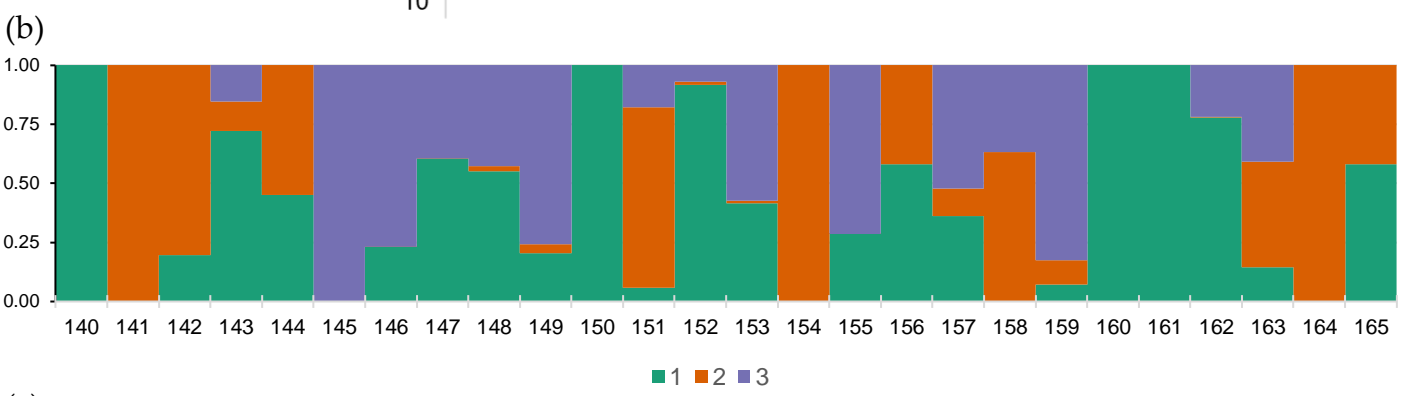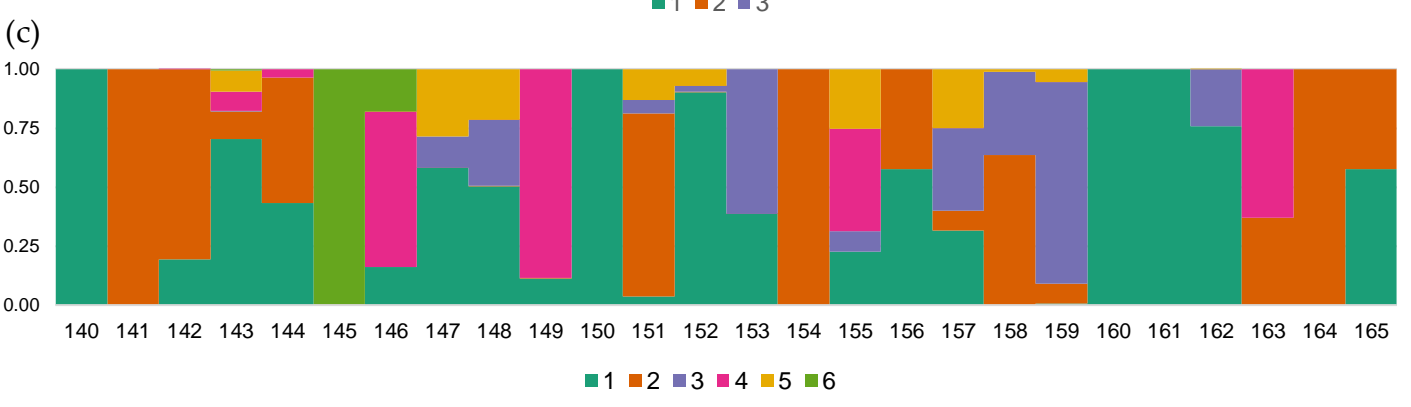

Supplement: Supplementary file 1 [file ijms-24-06547-s001.zip › Fig S5.pdf]

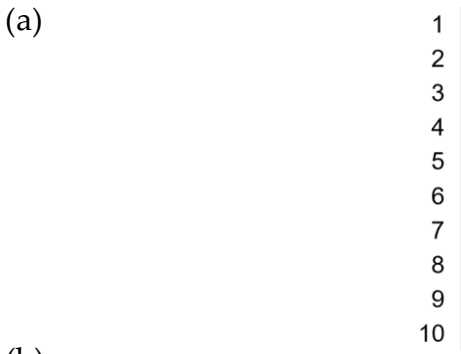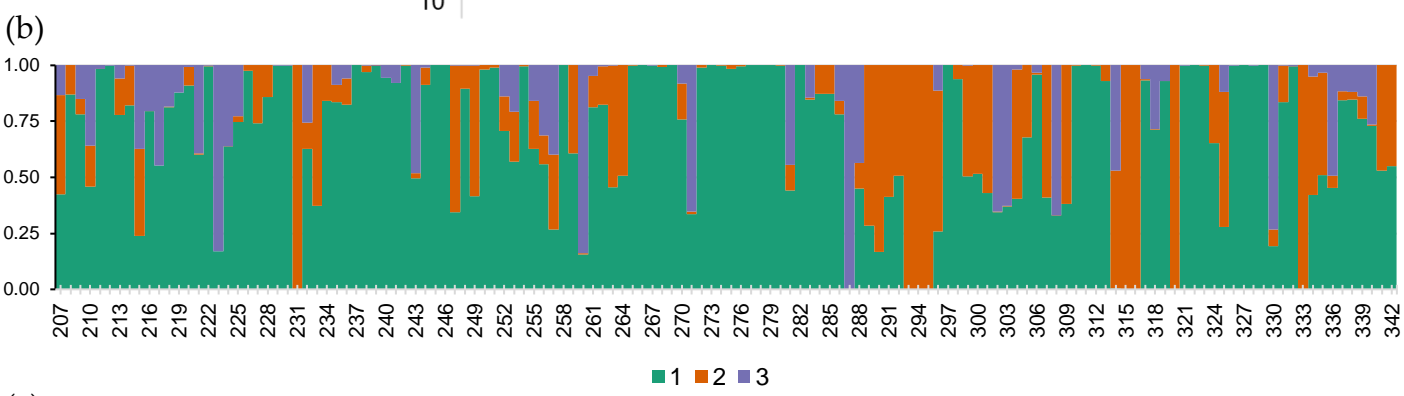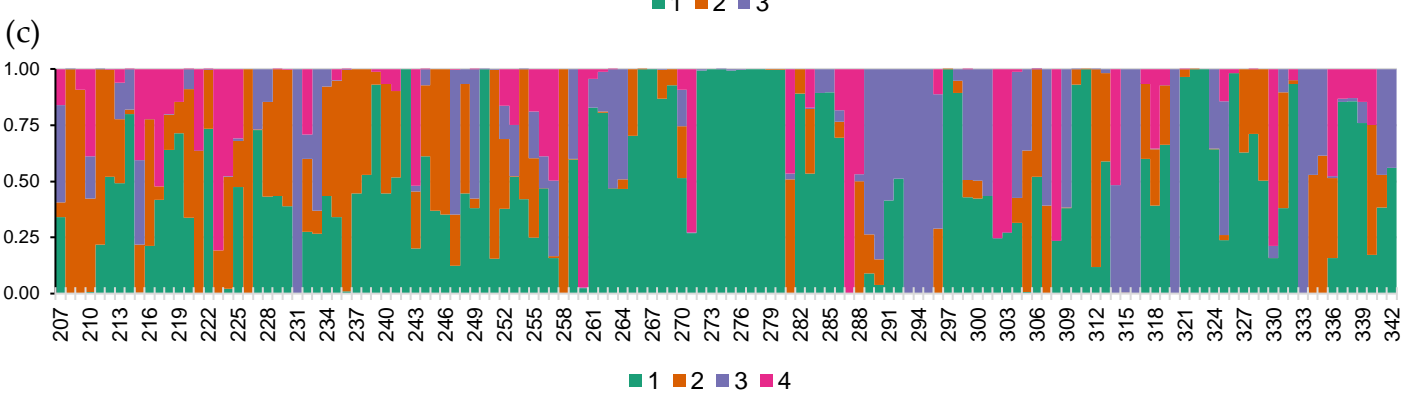

Supplement: Supplementary file 1 [file ijms-24-06547-s001.zip › Fig S6.pdf]

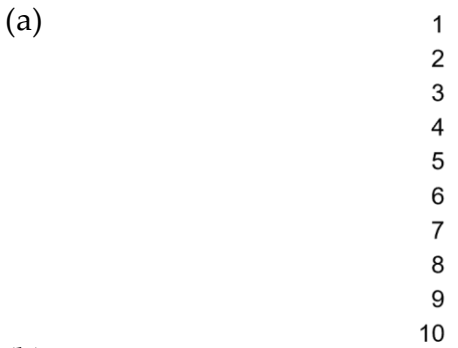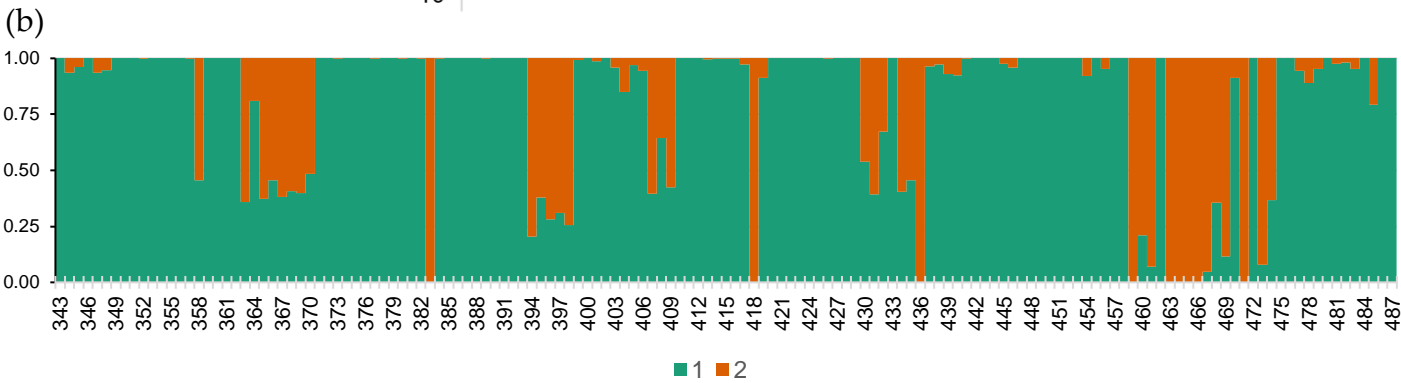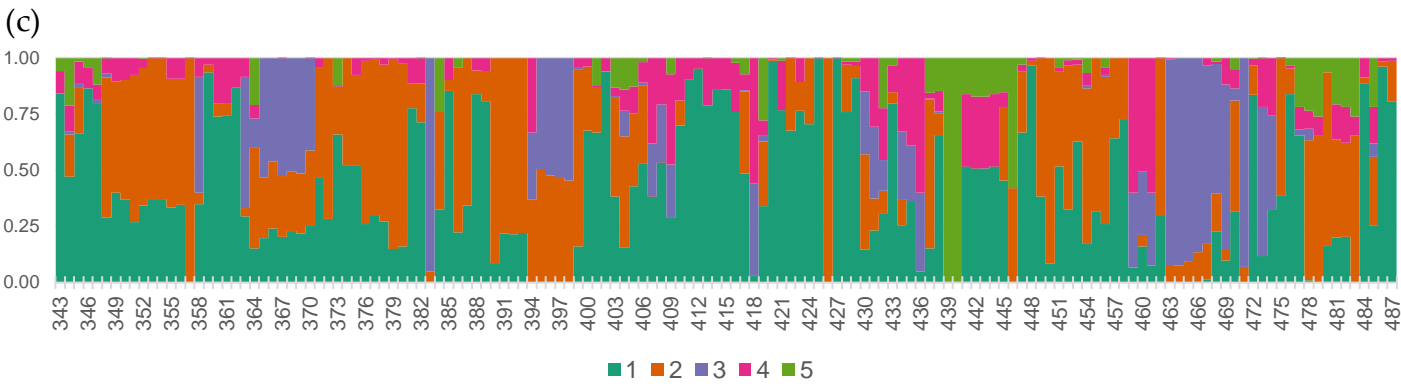

Supplement: Supplementary file 1 [file ijms-24-06547-s001.zip › Fig S7.pdf]

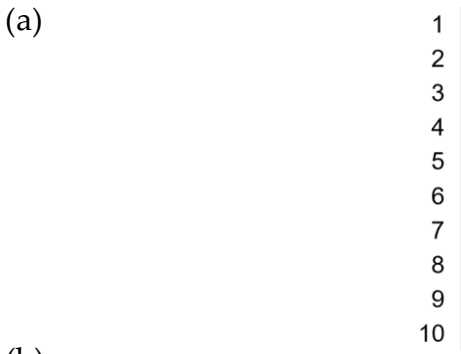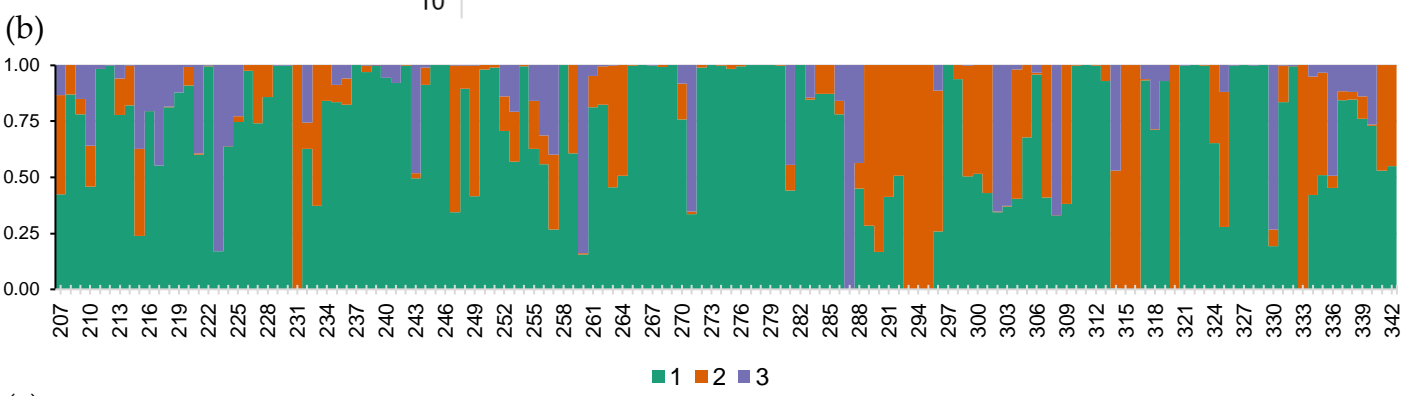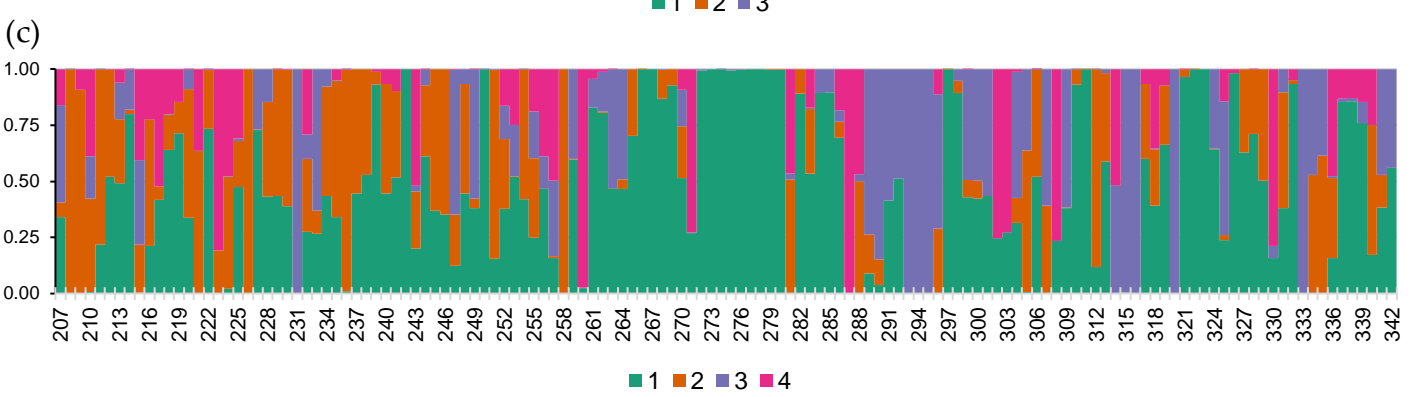

Supplement: Supplementary file 1 [file ijms-24-06547-s001.zip › Fig S8.pdf]
